# Diet and Nutrients in Gastrointestinal Chronic Diseases

**DOI:** 10.3390/nu12092693

**Published:** 2020-09-03

**Authors:** Antonio Corsello, Daniela Pugliese, Antonio Gasbarrini, Alessandro Armuzzi

**Affiliations:** 1 OU Internal Medicine and Gastroenterology, Fondazione Policlinico Universitario A. Gemelli IRCCS, 00168 Rome, Italy; danipug@libero.it (D.P.); antonio.gasbarrini@unicatt.it (A.G.); alessandro.armuzzi@policlinicogemelli.it (A.A.); 2 Istituto di Patologia Speciale Medica, Università Cattolica del Sacro Cuore, Largo F. Vito 1, 00168 Rome, Italy

**Keywords:** nutrition, gastrointestinal diseases, pediatrics, feeding disorders, functional gastrointestinal disorders, enteral nutrition, inflammatory bowel disease, irritable bowel syndrome

## Abstract

Diet and nutrition are known to play key roles in many chronic gastrointestinal diseases, regarding both pathogenesis and therapeutic possibilities. A strong correlation between symptomatology, disease activity and eating habits has been observed in many common diseases, both organic and functional, such as inflammatory bowel disease and irritable bowel syndrome. New different dietary approaches have been evaluated in order improve patients’ symptoms, modulating the type of sugars ingested, the daily amount of fats or the kind of metabolites produced in gut. Even if many clinical studies have been conducted to fully understand the impact of nutrition on the progression of disease, more studies are needed to test the most promising approaches for different diseases, in order to define useful guidelines for patients.

## 1. Introduction

Nutrition and its various facets play a fundamental role in the development and growth of the individual, both directly and indirectly modifying all physiological phenomena during the course of life [1].

Numerous studies have been conducted on the influence that different types of nutrition may have on the onset of chronic diseases at the level of the gastrointestinal system. As evidence of this, it has been seen that the epidemiology of some diseases can be very different depending on the region and the type of dominant diet in a given context [2]. Further studies, both on animals and humans [3], have been conducted on the role that the intestinal microbiota can have in the development of diseases, and how it can be directly influenced by both past and current food habits.

In addition to this, it has been observed that hypersensitivity to some types of food such us gluten can play a role in low-grade intestinal inflammation and in the increased intestinal permeability found in some patients [4].

Chronic intestinal pathologies, where a correlation between symptomatology, disease activity and eating habits has been observed, can be both inflammatory and dysfunctional, particularly in common diseases such as irritable bowel syndrome (IBS) [4], inflammatory bowel disease (IBD) [5], chronic constipation [6] and functional dyspepsia [7]. Another example, although less frequent, can be given by an organic pathology of unknown etiology such as eosinophilic esophagitis (EoE), where new dietary approaches seem very promising for future therapies [8]. Even if pathogenesis seems to be deeply different between functional diseases such as IBS and constipation and organic diseases such as IBD and EoE, food and diet seems to be strongly related to the evolution of disease and symptoms, and they are now often proposed as a common therapy approach.

The purpose of this review is to define the possible role of diet in the pathogenesis and management of most common gastrointestinal chronic diseases.

## 2. Diet, Microbiota and Inflammation

The human microbiota, composed not only of bacteria but also Archaea, fungi and viruses [9], changes deeply between different organs. Notably, in the intestine, it is characterized by an extremely dense and vast population, with around 10^11^ organisms and over 10^3^ different species per gram of feces [10]. Its estimated weight is 1kg, and its genes are about 100 times more than those of the human genome [11]. The four main “phyla” recognized and studied at the level of the intestinal microbiota are *Actinobacteria*, *Firmicutes*, *Proteobacteria* and *Bacteroidetes* [3]. They are in a symbiotic relationship with the organism, giving rise to multiple functions, both nutritional, such as food digestion and nutrient production for the organism, and immunologic, regulating mucosal immunity and protecting against the colonization of other hostile microorganisms [12,13,14,15].

Another fundamental characteristic is interindividual variability, which is caused by multiple factors, including genetic predisposition, environmental exposure, diet and lifestyle [16]. For this reason, the gut microbiota composition in the individual may change in response to intrinsic and extrinsic factors many times over the course of a lifetime. Indeed, an imbalance between protective and pathogenic bacteria can lead to a status called “dysbiosis”, which can cause an immune dysregulation of the organism and the pathogenesis of many diseases [17,18]. The role of dysbiotic microbiota in the pathogenesis of diseases is supported by animal models where colitis, or predisposition to other disease, can be transferred to wild-type mice using genetically-defined disease mice as gut microbiota donors [19].

Among the causes associated with the different epidemiologies of intestinal diseases and their increasing incidence, radical changes in modern lifestyle are among the most studied ones, e.g., the increased use of antibiotics and vaccines, improved healthcare and the reduction of parasitic infections, changes in eating habits, the wider use of refrigeration and the industrialization of food are just some of the main elements suspected [3,20,21,22,23,24]. This theory, which is called “the hygiene hypothesis”, argues that individuals in industrialized countries are exposed to fewer microbes in the early stages of life, leading to less immune tolerance and a greater possible dysregulation of immune-mediate responses [25].

The role that diet has in the development of the microbiota begins from the earliest stages of life, particularly with the introduction of solid foods, when the individual’s gut microbiota become increasingly stable and similar to those of adults [26,27,28]. Several studies have also evaluated the impact of diet on the gut microbiota of newborns, and have compared breast and formula feeding, with striking results; as an example, a significantly higher proportion of Bifidobacteria was found in breast fed infants compared to formula fed ones [29,30,31,32].

The main differences in the microbiota composition among groups of people exposed to significantly different diets have also been studied [33]. Groups that consume diets rich in animal proteins and fats and low in carbohydrates for a long time have been associated with high levels of *Bacteroides* and low levels of *Prevotella* [34,35]. By contrast, a simple carbohydrate-rich diet which is low in animal fats and proteins has been significantly associated with the opposite pattern [36,37].

In addition to this, it has also been seen that the microbiota of African children, whose diets are rich in plant derivatives and fiber, are profoundly different from those in a similar European population, whose diets are instead rich in carbohydrates, fats and proteins [3]. The different prevalence of diseases such as IBD between African and western countries could be linked to the different proportion of animal nutrients in the two diets. The “westernized” diet, rich in animal fats and protein and low in fiber, in fact, could be the reason for the altered gut microbiome, and therefore, for the increased risk for the development of diseases [38]. As an example of this, numerous epidemiological studies about multiple sclerosis suggested dietary traits as risk factors. The incidence of multiple sclerosis may be linked to the consumption of milk, animal fats and meat, as well as a high energy intake and obesity [39,40]. In contrast, diets containing high amounts of certain polyunsaturated fatty acids and plant fiber seem to decrease risk [38].

Another important feature of microbiota is linked to its plasticity in terms of responding quickly to diet changes. Fermentable carbohydrates, proteins and fats can modulate the balance between beneficial and pro-inflammatory microbes and metabolites such as SCFAs [41,42]. Nutrients that are not digested by host enzymes are then partly degraded in the cecum and colon through a process of fermentation performed by bacteria. An important product of fiber fermentation are SCFAs, which play multifactorial roles in energy regulation, host immunity and gut motility and absorption [43,44].

The modulation of gut microbiota can be also supplied through probiotics, which are “live microorganisms that, when administered in adequate quantities, can confer health benefits to the host” [45]. It has been suggested that probiotic preparations have positive health effects on the human intestine [46], modulating mucosal permeability, regulating neurotransmitter synthesis, strengthening the immune system and keeping away pathogens from the intestinal mucosa surface [47,48]. In particular, it has been suggested that *Bifidobacteria* and *Lactobacillus* species may compete with many pathogenic bacteria, and have been shown to be effective in the management of many gastrointestinal diseases, such as diarrhea (both infectious and antibiotic-related), pouchitis prevention, IBS, *Helicobacter pylori* and *Clostridium difficile* infections [47].

The association between diet, microbiota and inflammatory autoimmune diseases has been extensively studied in recent years [38,49,50], and the identification of inflammatory diseases relative to environmental risk factors remains a subject of intensive research. It has been shown in mice how segmented filamentous bacteria play a role in the development of arthritis and immune encephalomyelitis, a model for multiple sclerosis, inducing excessive T_H_17 cell response [51]. On the other hand, oral administration of the capsular polysaccharide A obtained from *Bacteroides fragilis* protected mice against the development of encephalomyelitis via conversion of CD4+ naive T cells into IL-10 producing T regulatory cells [52].

Many foods and nutrients such as milk, fats, carbohydrates, protein, fiber, fruit and vegetables have been studied as potential etiological factors in inflammatory diseases such as IBD, but the results have not succeeded in matching local or systemic inflammation with a specific food or food component [53,54]. However, recent systematic reviews note a possible predisposing role of a diet rich in animal protein and a protective effect of ω-3 polyunsaturated acids (n3-PUFA) in IBD [55,56,57,58,59].

Studies on the possible role of cow milk, fruit, juices and n3-PUFA in type 1 diabetes did not yield significant results [60,61,62,63]. Studies in multiple sclerosis, instead, suggested possible dietary habits as risk factors, such as the consumption of milk, meat and a high total energy intake with consequent obesity [64,65,66].

In addition to this, the association between obesity, metabolic syndrome and inflammatory gut disorders has been widely demonstrated [67,68]. White adipose tissue, in fact, releases many proinflammatory mediators such as TNF-α, IL-6, leptin and C-reactive protein, which contribute to chronic, low-grade, systemic inflammation in obese subjects [69,70]. Leptin in particular, a hormone which regulates body weight and energy metabolism, has been shown in leptin-receptor-deficient mice to be able to directly stimulate T lymphocyte proliferation and TH1 responses, which protect against lymphocytes apoptosis and promote inflammation through the production of IL-2 and IFN-γ [71].

Diets containing high amounts of saturated fats, dairy products and simple refined carbohydrates, and a lower quantity of vegetables, cereals and fibers have also been shown to increase the risk of diseases such as obesity, hypertension and chronic kidney disease [72,73,74], promote intestinal inflammation and modify the microbiota composition [75]. In addition, these diets are low in micronutrients with anti-inflammatory and antioxidant proprieties [54]. It is possible to consider these kind of diets as one of the major environmental factors linked to the rising incidence of autoimmune inflammatory diseases in western countries [66]. All these observations are just some of the reasons why gut dysbiosis and diet could be heavily involved in the development of various human diseases, and why further studies of their possible associations are required.

## 3. IBS

IBS is a functional gastrointestinal disease with an estimated global prevalence of between 5 and 20% [76]. The syndrome is defined by the Rome IV criteria [77] as a recurring abdominal pain for at least 4 days a month in the past two months, associated with one or more symptoms of changes in the frequency or characteristics of the stool. Its pathophysiology is still not entirely clear, but it seems that alterations of the microbiota, hypersensitivity and intolerance to some foods, increased intestinal permeability, a low-grade inflammation in the mucosa and the protracted use of antibiotics may be some of the most important elements in the development of symptoms [78].

Over 2/3 of IBS patients are convinced that their symptoms are associated with the intake of certain foods such as milk and its derivatives [79], leading some avoid such foods, with an estimated 12% risk of long-term nutritional deficits [80]. Nevertheless, no specific significant correlation was found in patients who reported certain foods or nutrients as triggers of intestinal symptoms [81,82]. Despite this, some foods seem to be associated with the generation of symptoms [4,83].

### 3.1. Fermentable Oligosaccharides, Disaccharides, Monosaccharides and Polyols (FODMAP)

Many proposals have been made to try to explain how nutrition can influence IBS, including the reduced absorption of fiber and carbohydrates, the comorbidity of diseases such as obesity, and the presence of food intolerances or allergies [78]. Among the various hypotheses and studies conducted, the most promising are those that have linked the trigger of gastrointestinal symptoms to the intake of foods containing FODMAPs, which are less readily absorbed [84]. Mostly contained in some fruits, legumes, dairy products and artificial sweeteners, they can exacerbate symptoms due to their fermentation and osmotic effects in the lumen. These carbohydrates arrive at the level of the colon, where they induce the production of gas following the fermentation caused by the bacterial colic flora, with a consequent luminal distension. Recent studies have also shown how an interaction between FODMAPs and the microbiota can act on intestinal stem cells and the number of gastrointestinal endocrine cells, as they have been shown to be associated with an increased level of their respective markers Neurogenin 3 and Chromogranin A [85].

A diet based on “low-FODMAP” foods (Table 1) seems to induce a significant improvement in symptoms in 2/3 of IBS patients, with response times as low as 1–2 weeks [86]. Many observational studies have been conducted on the low-FODMAP diet in recent years, showing that this diet can significantly reduce abdominal pain, flatulence and diarrhea. On the other hand, no clear evidence was found in relation to the improvement of symptoms on the basis of an increase in the total fiber quantity and reduced gluten intake [79].

Another important point is that the low-FODMAP diet, in contrast to other exclusion diets, allows the patient to consume foods from each of the core food groups, minimizing the effect on nutrition adequacy when appropriately implemented [87].

A recent meta-analysis on the effects that a diet low in FODMAPs can have in reducing symptoms associated with functional gastrointestinal disorders found significant results in the improvement of individual gastrointestinal symptoms among IBS patients, such as abdominal bloating (77% of patients, *p* < 0.001), flatulence (76%, *p* = 0.001), abdominal pain (71%, *p* < 0.001), diarrhea (70%, *p* = 0.001), nausea (65%, *p* = 0.001) and constipation (44%, *p* = 0.01) [88].

### 3.2. Dietary Approach

Currently, and particularly in children, an exclusion diet is not indicated as a first-line approach for the management of IBS [87]. Proper nutrition (three meals a day at regular times), good hydration (1.5–2 L per day) and a limitation of potential disease triggers such as alcohol, caffeine, spicy and fatty foods are the main recommendations [79]. Alcohol has indeed been shown to alter motility, absorption and intestinal permeability [88]. Caffeine increases gastric acid secretion and colic motility [89]. The capsaicin contained in spicy foods accelerates intestinal transit and stimulates visceral pain sensations [90]. The consumption of fats (>50 g per day) is associated with reduced motility of the small intestine and increased sensation of distension of the rectum and should be avoided [91].

However, a second-line approach should be considered in cases where symptoms persist despite correct eating habits, and in this scenario, a low FODMAP diet seems to be the most suitable approach, based on the current evidence [89].

## 4. Chronic Constipation

Chronic constipation has been defined as the reduction in the number of evacuations to less than 3 per week for a protracted period of time [90], with an overall estimated prevalence of 16% among the general population [91]. Common causes of chronic constipation include a lack of fiber, linked to inadequate consumption vegetables and fruits, and insufficient fluid intake. Fortunately, functional food-related constipation is usually not a serious problem and can be controlled and treated by correcting eating habits and lifestyle [6].

Three different types of primary chronic constipation have been described, which show substantial overlap among each other and other diseases such as IBS. These three types are rectal evacuation disorders, slow transit constipation and normal transit constipation; the latter seems to represent the largest group [92].

Nevertheless, the pathogenesis of functional constipation is usually multifactorial, with interplay among the type of diet, genetic predisposition, intestinal motility, structure and absorption, as well as psychological, biological, and pharmaceutical factors [93].

Among the studies that have been conducted in this field, it has been shown that the response to food in patients with slow transit constipation is characterized by shorter contractile activity in all the three-colon segments and the rectum, and significantly fewer high-amplitude propagated contractions [94].

A case-control study that evaluated 10 healthy controls and 10 patients with constipation in response to a liquid meal and intracolonic stimulant laxative bisacodyl infusion [95] showed increased motility in healthy participants, but not in patients with constipation, who showed instead a longer duration and decreased number of high amplitude propagating contractions. A reduction in the density of excitatory nerve fibers (tachykinin and enkephalin) was found in the colonic circular muscle of patients with slow transit constipation, whereas innervation of all the other layers was normal [96]. These abnormalities in neurotransmitters could contribute to dysmotility and the development of symptoms.

A systematic review of six different randomized controlled trials found that soluble fibers (particularly psyllium, at least 10 g daily, or ispaghula) led to improvements in global symptoms (86.5% vs. 47.4%), straining (55.6% vs. 28.6%), pain on defecation, stool consistency, an increase in the mean number of stools per week (an average of 3.8 stools per week after therapy compared with 2.9 per week at baseline) and a reduction in the number of days between stools [97]. The mechanisms that link the ingestion of soluble fibers to a laxative effect are mainly irritation of the mucosa, which stimulates water and mucus secretion, and the property of resisting dehydration and carrying water to the colon, changing stool consistency [92]. The absence of a response to dietary fiber supplementation may suggest that there are additional factors contributing to constipation.

Even if the management of chronic constipation is not always simple, due to the possible different pathophysiology, as with IBS management, studies have indicated that a soluble fiber-rich diet can increase stool weight, resulting in a reduction in colon transit time, while a low-fiber diet may induce constipation [97].

## 5. Functional Dyspepsia

Functional dyspepsia is a debilitating functional gastrointestinal disorder characterized by early satiety, postprandial fullness or epigastric pain related to meals, which affects up to 20% of the western population [98]. It includes different clinical entities, and patients are generally assigned to one of two subtypes: postprandial distress syndrome (PDS), the symptoms of which are strictly related to food intake, and epigastric pain syndrome (EPS) [99]. These conditions are characterized by various gastrointestinal symptoms in the upper part of the abdomen, and the absence of an organic underlying pathology [7].

Although symptoms occur after food consumption, a few clinical trials have formally evaluated dietary interventions for the management of functional dyspepsia [100]. These studies suggest that associations may exist between symptoms of functional dyspepsia and dietary variables such as total energy and food volume intake, meal frequency and psychological conditioning related to specific foods [101].

The finding of duodenal eosinophilia in patients with functional dyspepsia suggests that food antigens may play a role in the disease, perhaps through stimulation of mucosal immunity and increasing the intestinal permeability [102].

Many foods are reported by patients to be associated with the onset of symptoms (e.g., diets rich in fermentable carbohydrates, sugary drinks, fruit, milk, wheat). Although the association between FODMAPs and irritable bowel syndrome is well recognized, some authors highlight the need for further investigation into FODMAP-rich nutrients related to other conditions, including functional dyspepsia [103]. Of particular interest are investigations on hypersensitivity to gas production and osmotic pressure within the upper digestive tract.

Gluten (and other wheat-related proteins) and FODMAPs are hypothesized to be triggers of symptoms in irritable bowel syndrome [99]. Apart from gluten, wheat contains other low-weight proteins called α-amylase/trypsin inhibitors (ATIs), which seem to be related to a gastrointestinal and systemic disease named “non-celiac gluten hypersensitivity”. In this condition, the consumption of wheat or gluten-containing foods results in symptoms that overlap with FD, leading to the hypothesis that this disease may be a subtype of FD [104]. ATIs seems to be able to stimulate innate immune cells via the activation of toll-like receptor 4, which induces the release of pro-inflammatory cytokines and chemokines [105]. Wheat-containing foods have been implicated in inducing dyspepsia symptoms in five recent studies, two of which were specific to gluten and randomized [106,107,108,109,110]. Endpoints of these studies were evaluated through physical and mental health questionnaires and visual analogue scales referring to their symptoms. Although the implementation of a gluten-free diet in both studies clearly demonstrated a reduction in symptoms in an average of 75% of patients, the elimination of wheat, barley and rye also substantially reduced the FODMAP content of these diets, potentially influencing the results [109].

Even if there is no standardized approach to the dietary management of functional dyspepsia, current dietary suggestions focus on eating more frequent and smaller meals, based on the evidence that a high meal volume and gastric distension could be implicated in triggering symptoms, and on “low-fat” diets [111]. It has been demonstrated that fats could exacerbate the symptoms of dyspepsia through delayed gastric emptying and hypersensitivity to gastrointestinal hormones [112].

## 6. IBD

IBD is a group of chronic diseases with a multifactorial etiology, characterized by a local and systemic inflammatory pathological process and a natural history that presents phases of exacerbation and remission, with the recurrence and progression of symptoms of intestinal damage. The IBD spectrum includes two main clinical phenotypes: Crohn’s disease (CD) and ulcerative colitis (UC) [113].

Many studies indicate a possible role of diet in the development of IBD [38]. It has also been observed that the microbiota of IBD patients are very different from those of healthy individuals, and that this can affect the digestion and metabolism of many foods, such as fiber and SCFAs [3].

Among the various foods studied regarding a possible correlation with IBD, particular attention was paid to the increased intake of animal proteins and polyunsaturated fats (n3-PUFA) [55,56,114].

### 6.1. Animal Proteins

The main mechanism linked to the possible association between animal protein intake and IBD is the malabsorption of the heme and amino acids contained in animal proteins, which are not absorbed by the small bowel and, therefore, reach the colonic lumen, where they are metabolized by the microflora, with the consequent production of toxic molecules like hydrogen sulfide, phenol and ammonium [21,115], which, in turn, reduce the abundance of “anti-inflammatory” bacteria like *Roseburia* and *Eubacterium rectale*, which are capable of producing butyrate [116].

Additional mechanisms, such as the impact of the cooking process upon inflammatory response, and endothelial damage, through the production of carcinogenic molecules (polycyclic aromatic hydrocarbon, heterocyclic amines and acrylamide), also require examination [117].

### 6.2. Animal Fats

Furthermore, strong correlations between the consumption of red meat and IBD have been found in relation to high saturated fatty acid (PUFA) intake [118]. As proof of this, a diet rich in animal fats seems to promote dysbiosis and intestinal inflammation, with the consequent predisposition to developing IBD [119]. The role of PUFAs in IBD can be explained through the eicosanoids produced by n3- and n6- PUFA, which are precursors of several pro-inflammatory and anti-inflammatory molecules, regulating the production of molecules such as prostaglandin E2 and thromboxane B2 [120].

A high fat diet should be also avoided by IBD patients due to the fact that it could lead to an accumulation of bile acids (like deoxycholic acid) that could inhibit *Bacteroidetes* and *Firmicutes* phyla growth and lead to dysbiosis [54]. A recent cross-over study [121] demonstrated that a low-fat diet reduces markers of inflammation and dysbiosis and improves quality of life in patients with ulcerative colitis. In particular, it has been shown that low-fat diets increase the proportion of *Bacteroidetes* in the microbiota from 14.6% to 24.02% (*p* = 0.015) and decrease that of *Actinobacteria* from 13.69% to 7.82% (*p* = 0.017).

### 6.3. Fibers and Sugars

High fiber and fruit consumption have been associated with a lower risk of developing IBD [122]. Fibers are present in vegetables, cereals, fruits and legumes, and they are resistant to digestion by the human gut due to the lack of fiber-specific enzymes; as such, they are not absorbed [123]. Furthermore, fibers are fermented by the bacteria in the colon [124], and their possible protective role against IBD is linked to their osmotic capacity to reduce intestinal transit time [125] and to the promotion of the proliferation of bacteria that prevent dysbiosis and mucosal inflammation.

However, high fiber diets can worsen gastrointestinal symptoms such as abdominal pain and diarrhea, and for this reason, it has been recommended that patients in remission consume a diet with low levels of insoluble fibers, such as those contained in vegetables like zucchini, carrots and eggplants [126].

### 6.4. Micronutrients Deficiency and Supply

IBD usually leads patients to suffer from vitamin and mineral deficiencies, due to the malabsorption linked to inflammation and the reduced intake of food [127]. For this reason, diet or supplementation adjustment strategies are generally suggested. The main vitamin deficiencies observed in IBD-patients are water-soluble B9 and B12, and fat-soluble vitamins A and D [128]. B9 and B12 vitamin are absorbed in the duodenum, jejunum and ileum, areas which are often damaged by IBD [128]. It has been demonstrated in experimental models that vitamin A supplementation attenuates intestinal inflammation [75]. As an antioxidant, vitamins A seems to have a protective role against free radicals and oxidative damage, which are significant elements in the inflammation processes [129]. Vitamin D improves intestinal defense mechanisms and regulates the adaptive and innate immune systems, preventing microbial proliferation [130]. Nonetheless, the efficacy of vitamins in the treatment of IBD is still not totally clear, and clinical studies have shown ambiguous results. Two studies were conducted on the use of vitamin D3 supplementation in a cohort of patients, and while both showed a reduced risk, neither yielded significant results [131,132].

The main mineral micronutrient deficiencies observed in IBD patients are iron and zinc [128]. Iron deficiency is a common problem and the main cause of anemia in IBD patients, with an estimated prevalence of more than 25% [133]. An iron-rich diet or supplementation is therefore often used as a therapy strategy. A deficiency of zinc, which is a key enzyme cofactor in cellular immunity, growth and wound healing, has been found in 15% to 40% of patients, and has been associated with chronic diarrhea and surgery or disease complications in IBD patients [134].

### 6.5. Dietary Therapies

Another hint regarding how nutrition can be closely correlated with disease activity is the fact that exclusive enteral nutrition (EEN) is capable of inducing clinical and endoscopic remission in pediatric patients; although the mechanism for this it is not well known, it seems that it may be due to the reduced exposure to risk factors and to the anti-inflammatory activity of some nutrients contained in the formula [135].

EEN is the first-choice treatment for pediatric patients to obtain an improvement in nutritional status and to induce remission of luminal CD, with an observed efficacy of more than 90% [136]. The EEN approach is usually based on the exclusive administration of a polymeric formula for a period ranging from 6 to 8 weeks [136]. A meta-analysis conducted on pediatric studies showed that EEN has the same efficacy as corticosteroids in inducing remission in patients with active CD, with the advantage of reducing the side effects [137]. In addition to its efficacy in inducing remission, some studies support the role of EEN in inducing Mucosal Healing; even if the mechanisms are not yet understood, it has been shown that EEN is as effective as Infliximab inhibitor of tumor necrosis factor (TNF)-α in the maintenance of the function of the intestinal barrier, and significantly better than hydrocortisone [138]. Based upon this evidence, the latest guidelines on pediatric CD therapeutic management state, for the first time, that EEN, rather than steroid therapy, should be used as first-line induction therapy in CD [139].

EEN use is now widely being studied also in adults, where it seems to be promising. A recent study conducted on 38 adult patients (aged from 16 to 40 years) with active disease showed that the 84% of patients who completed 2 weeks of EEN had significant improvements in terms of both symptoms (*p* = 0.003), C-reactive protein (CRP) (*p* = 0.005) and fecal calprotectin (*p* = 0.028) levels [140]. EEN could therefore be evidence of how a “healthy” diet can induce remission in patients with IBD.

Another approach that was recently evaluated through a randomized controlled trial is the CD treatment-with-eating diet (CD-TREAT), a personalized and tolerable diet with comparable composition to EEN, based on the exclusion of certain dietary components (gluten, lactose and alcohol) and the inclusion of others (macronutrients, vitamins, minerals and fibers) using ordinary food [141]. The study was conducted on healthy volunteers, pediatric IBD patients and animal murine models, in order to evaluate the effects of the diet on gut microbiome composition, inflammation and clinical response. CD-TREAT achieved greater compliance than EEN, and positive effects were found on fecal calprotectin (mean decrease of 918 ± 555 mg/kg, *p* = 0.002) and gut microbiome composition (lower levels of butyrate, propionate and SCFAs). Additionally, promising results were observed regarding clinical response, which occurred in 80% of participants [142]. CD-TREAT seems to be a potential substitute for EEN, particularly in adults for whom EEN tolerability is low (mainly related to taste fatigue and its unpalatability), and a good option for long-term dietary maintenance therapy.

A significant percentage of IBD patients also suffer from symptomatic symptoms similar to those of functional irritable bowel, even when on clinical remission [143]. The low-FODMAP diet has been associated with improved symptoms in many of these patients [54]. However, the influence of the low FODMAP diet on the microbiome, metabolism and inflammation in patients with IBD is still unclear. Reduced concentrations of inflammatory cytokines, such as TNF-α, IL-6 and IL-8, have also been observed, demonstrating a possible anti-inflammatory effect of dietary fiber, probably through their influence on the microbiota [122,144].

Anew diet, the Crohn’s Disease Exclusion Diet (CDED) (Table 2) [135,145] has also been formulated. CDED is a structured diet based on the hypothesis that a Western diet rich in fats and refined sugars may cause inflammation and altered permeability of the mucosal barrier. It is based on the exclusion of animal fats, dairy products, gluten and all processed foods that contain additives and emulsifiers [146]. A recent randomized controlled trial which evaluated 74 patients with mild to moderate CD was conducted, comparing a combo therapy made of partial enteral nutrition (PEN) (50% of daily calories provided as a formula) and CDED to EEN for 12 weeks. The study showed success in achieving induction of clinical remission of CDED. Furthermore, remission was obtained in 70% of children and 69% of adults, and a normalization of previously elevated CRP values occurred in 70% of patients in remission [147]. The primary endpoint of the study, as a marker of its effectiveness, was patient’s tolerance to the diet. Tolerance was significantly different among the two groups, favoring CDED + PEN over EEN: 97.5% vs. 73.7% (*p* = 0.002). Significant differences in clinical response and inflammation (using Pediatric Crohn Disease Activity Index (PCDAI) and CRP values) was also found at week 12 between the two groups, showing how a PEN, if associated with dietary therapy, can better improve both symptoms and remission (PCDAI <10 achieved by 75.6% of patients on CDED + PEN vs. 45.1% of EEN ones, *p* = 0.01; Normal CRP occurred in 75.9% of patients on CDED + PEN vs. 47.6% of EEN ones, *p* = 0.04). This multicentric study showed how a dietary rationalized option, which combines food and “traditional” therapy, can globally improve patient conditions and clinical response.

Although these diets are usually prescribed for short-term use, many patients continue to use them for a long period of time. Even if clinical studies have failed to find any significant differences in the severity of long-term symptoms, the number of complications or the need for hospitalization or surgery between patients who use restrictive diets and those who consume an unrestricted diet, evidence suggests that a balanced diet can help in maintaining the remission of disease and improving quality of life [148].

**Figure 1:**
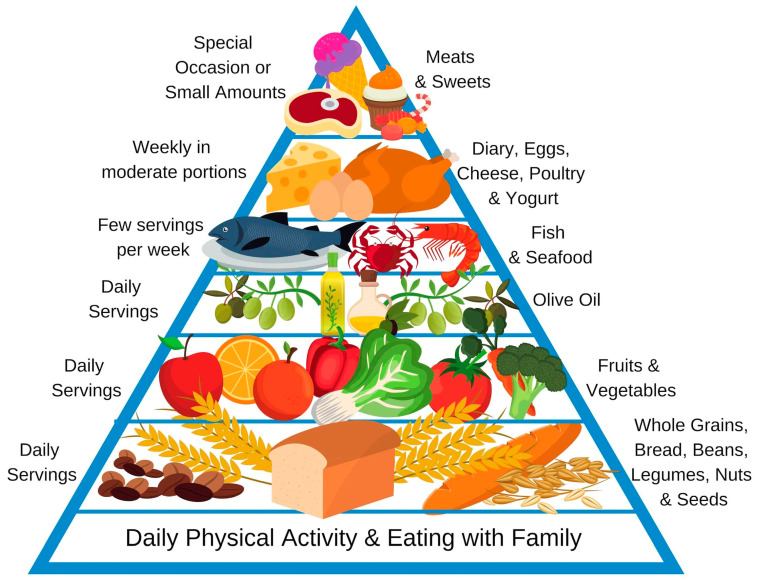
Mediterranean diet’s standardized food pyramid.

One long-term dietary pattern that could be useful for the management of intestinal diseases such as IBD (and particularly under remission) is the Mediterranean diet. It is characterized by a high consumption of olive oil and fish, which are rich in mono and polyunsaturated fatty acids [149], fruits, whole grains and vegetables, in order to afford adequate intake of fiber (i.e., about 30 g per day according to the WHO suggestions [150]). This diet is based on the daily or weekly consumption of specific food groups according to the standardized food pyramid (Figure 1) [151]. The Mediterranean diet has been shown to have a potentially positive effect on the composition of the microbiota, helping to return to eubiosis and reducing the production of pro-inflammatory factors such as NF-kB [152], and a recent study demonstrated that a good adherence to this diet is significantly associated with higher levels of total SCFAs in the gut [153]. Furthermore, the possible role of the Mediterranean diet in the modulation of gene expression in IBD patients has been studied in the laboratory [154].

## 7. Eosinophilic Esophagitis

EoE is a chronic immune-mediated inflammatory disease of the esophagus associated with a deposit of eosinophils in the esophageal wall. The causes are still unknown, but inflammation may depend on the combination of genetic and environmental factors. Often, this condition is associated with allergic syndromes induced by food antigens [155]. EoE can occur in both children and adults, and is more predominant among males. Dysphagia, gastroesophageal reflux and heartburn are among the most frequent symptoms. If untreated, inflammation of the esophagus can lead to lumen stenosis.

Among nonpharmacological therapies for the management of EoE in adults and children, the modification of diet may be the most important. It is based on the role that dietary antigens play in the pathogenesis of this disease. Currently, there are two main choices for initial diet therapy for EoE: elementary diets, based on the administration of simple amino acids, and elimination diets [8]. A group of 10 children with EoE were evaluated through an elementary diet [156]. Patients had a resolution or improvement in symptoms for a minimum of 6 weeks and showed a significant reduction in esophageal eosinophilia. In addition to this, symptoms recurred when the proteins were reintroduced, which implies that food allergies were responsible for inflammation esophageal.

A meta-analysis found that elementary diets were effective in 90.8% of cases, and that they were superior to both the six-food elimination diet (SFED), which was effective in 72.1% of cases, and to elimination diets guided by specific allergy tests, which were effective in 45.5% of cases [157]. However, this dietary strategy seems difficult in clinical practice; its main limitations include the poor compliance of the formula (which requires a nasogastric tube in most children, with consequent adherence by only a third of patients), psychological and social disorders, changes in the quality of life due to the lack of variety of foods, and high cost, since these diets are not universally covered by health systems [156]. Although the goal of the diet is to eliminate exposure to specific triggers, a serious defect is the period of time and the number of endoscopies necessary to identify specific triggering factors during the food reintroduction phase. A retrospective study was conducted in 2006, including 60 children with EoE and comparing the SFED, in which proteins from cow’s milk, soy, wheat, eggs, peanuts and crustaceans were eliminated, with the elementary diet; after 6 weeks, clinical and histological remission was observed in 74% of patients with SFED, a result that was not inferior to the elementary diet but which had better acceptance, adherence and cost [158].

## 8. Conclusions

Even if studies are not always clear and unambiguous, it is important to not underestimate the role that nutrition and diet can have, not only in the development of chronic gastrointestinal diseases, but also in maintaining the remission of symptoms and, most importantly, in improving the quality of life of patients. The exact role that diet can have in each gastrointestinal disease is not totally understood, but there is much evidence in support of the notion that food and controlled nutrition can prevent and modulate symptoms.

Among all diseases and the different approaches which have been evaluated in recent years, a possible link between “westernized diet” and the worsening of symptoms seems to be evident, and the necessity of a controlled intake of animal fats and refined sugars is needed in patients who suffer from any functional or organic gastrointestinal disease.

New, strict dietary approaches and novel treatment options are being more widely evaluated for common diseases like IBD and IBS, and some promising results have been found from exclusion diets such as “low-FODMAP”, CD-TREAT and CDED. The wide variability of some studies’ results can be attributed both to the different study designs (mostly retrospective or conducted in small cohorts of patients) and to the great heterogeneity of the symptoms of gastrointestinal diseases.

Even if strong evidence from recent meta-analyses supports the fact that rationalized dietary options can improve patients conditions, at least in the medium-term, more studies are needed to test the most promising dietary approaches for each disease in order to evaluate the positive and negative impact that different foods and diets can have on the modulation of gut homeostasis and microbiota, the effective long-term dietary adherence, and other possible implications on the health of patients.

## Figures and Tables

**Table 1:** Food included or excluded in the low-FODMAPs diet.

Type of Food	Low-FODMAP (Include)	High-FODMAP (Exclude)
Vegetables	Carrots, celery, corn, bean sprouts, bell pepper, broccoli, cucumber, eggplant, green bean, lettuce, potato, spinach, tomato, zucchini	Brussels sprouts, asparagus, avocado, beetroot, cauliflower, cabbage, garlic, leek, mushroom, onion, pea shallot, snow pea, sweet corn, sweet potato
Fruit	Bananas, strawberry, raspberry, blueberry, orange, mandarin, cantaloupe, grapes, melons, lemon, lime, kiwi, passion fruit	Apples, applesauce, apricots, blackberries, cherries, nectarines, pears, peach, plum, prune, watermelon, grapefruit, dried fruit
Grains	Rice, oats	Wheat, rye
Dairy	Lactose-free yoghurt and milk; almond, coconut, rice or soy “milk”, hard cheese, low-lactose cheese	Cow, goat and sheep milk, buttermilk, soymilk, soft cheese cream and ice cream
Meat	Beef, chicken, lamb, pork	Sausages, processed meat
Drinks	Fruit and vegetable juices from permitted foods, wine	Soft drinks, sports drinks, juices from unpermitted foods, beer

**Table 2:** CDED dietary instructions during induction phase (first 6 weeks).

Mandatory Daily Foods and Quantities	Disallowed Foods
Fresh Chicken breast 150–200 g/d	Dairy
2 Eggs/d	Animal fat
2 Bananas/d	Wheat
1 Fresh Apple/d	Emulsifiers
2 Potatoes/d	Artificial Sweeteners
**Allowed Foods Daily**	Other cuts or parts of chicken
Fresh Strawberries	Other sources animal or soy protein
Fresh Melon (1 slice)	Carrageenans
Rice flour	Maltodextrins (and sucralose)
White rice and rice noodles (unlimited)	Sulfite containing foods
2 Tomatoes (additional allowed for cooking)	Xanthan gum
2 Cucumbers (medium size)	Packaged or frozen precooked foods
2 Avocado halves	doughs, baked goods
1 Carrot	Frozen, canned fruits and vegetables
Spinach 1 cup uncooked leaves	Oral Iron supplements
Lettuce (3 leaves)	Soy or Gluten-free products
Onion	Ready to use sauces, dressings, margarine, butter
Fresh green herbs (basil, parsley, coriander, rosemary, thyme, mint, dill)	Vinegar, soy sauce, ketchup, mayonnaise
	Alcoholic beverages, soft drinks, juices
1 glass of squeezed orange juice from fresh oranges	Deep-fried or oily foods
Water, sparkling water	
Salt, pepper, paprika, cinnamon, cumin	
3 tablespoons honey	
4 teaspoons sugar	
Fresh ginger and garlic cloves, lemons	

## References

[1] Lee D., Albenberg L., Compher C., Baldassano R., Piccoli D., Lewis J.D., Wu G.D. (2015). Diet in the Pathogenesis and Treatment of Inflammatory Diseases. Gastroenterology.

[2] De Filippo C., Di Paola M., Ramazzotti M., Albanese D., Pieraccini G., Banci E., Miglietta F., Cavalieri D., Lionetti P. (2017). Diet, Environments, and Gut Microbiota. A Preliminary Investigation in Children Living in Rural and Urban Burkina Faso and Italy. Front. Microbiol..

[3] Wu G.D., Bushmanc F.D., Lewis J.D. (2013). Diet, the human gut microbiota, and IBD. Anaerobe.

[4] Volta U., Pinto-Sanchez M.I., Boschetti E., Caio G., De Giorgio R., Verdu E.F. (2016). Dietary Triggers in Irritable Bowel Syndrome: Is There a Role for Gluten?. J. Neurogastroenterol. Motil..

[5] Chapman-Kiddell C.A., Davies P.S.W., Gillen L., Radford-Smith G.L. (2010). Role of diet in the development of inflammatory bowel disease. Inflamm. Bowel Dis..

[6] Tucker D.M., Sandstead H.H., Logan G.M.J., Klevay L.M., Mahalko J., Johnson L.K., Inman L., Inglett G.E. (1981). Dietary fiber and personality factors as determinants of stool output. Gastroenterology.

[7] Duncanson K.R., Talley N.J., Walker M.M., Burrows T.L. (2018). Food and functional dyspepsia: A systematic review. J. Hum. Nutr. Diet..

[8] Gómez-Aldana A., Jaramillo-Santos M., Delgado A., Jaramillo C., Lúquez-Mindiola A. (2019). Eosinophilic esophagitis: Current concepts in diagnosis and treatment. World J. Gastroenterol..

[9] Scarpellini E., Ianiro G., Attili F., Bassanelli C., De Santis A., Gasbarrini A. (2015). The human gut microbiota and virome: Potential therapeutic implications. Dig. Liver Dis..

[10] Garrett W.S., Lord G.M., Punit S., Lugo-Villarino G., Mazmanian S.K.K., Ito S., Glickman J.N., Glimcher L.H. (2007). Communicable Ulcerative Colitis Induced by T-bet Deficiency in the Innate Immune System. Cell.

[11] Rajilić-Stojanović M., De Vos W.M. (2014). The first 1000 cultured species of the human gastrointestinal microbiota. FEMS Microbiol. Rev..

[12] Podolsky D.K. (2002). Inflammatory bowel disease. N. Engl. J. Med..

[13] Aleksandrova K., Romero-Mosquera B., Hernandez V. (2017). Diet, gut microbiome and epigenetics: Emerging links with inflammatory bowel diseases and prospects for management and prevention. Nutrients.

[14] Dinan T.G., Cryan J.F. (2017). The Microbiome-Gut-Brain Axis in Health and Disease. Gastroenterol. Clin. N. Am..

[15] Huttenhower C., Gevers D., Knight R., Abubucker S., Badger J.H., Chinwalla A.T., Creasy H.H., Earl A.M., Fitzgerald M.G., Fulton R.S. (2012). Structure, function and diversity of the healthy human microbiome. Nature.

[16] Healey G.R., Murphy R., Brough L., Butts C.A., Coad J. (2017). Interindividual variability in gut microbiota and host response to dietary interventions. Nutr. Rev..

[17] Viggiano D., Ianiro G., Vanella G., Bibbò S., Bruno G., Simeone G., Mele G. (2015). Gut barrier in health and disease: Focus on childhood. Eur. Rev. Med. Pharmacol. Sci..

[18] Lopetuso L.R., Scaldaferri F., Bruno G., Petito V., Franceschi F., Gasbarrini A. (2015). The therapeutic management of gut barrier leaking: The emerging role for mucosal barrier protectors. Eur. Rev. Med. Pharmacol. Sci..

[19] Elinav E., Strowig T., Kau A.L., Henao-Mejia J., Thaiss C.A., Booth C.J., Peaper D.R., Bertin J., Eisenbarth S.C., Gordon J.I. (2011). NLRP6 inflammasome regulates colonic microbial ecology and risk for colitis. Cell.

[20] Ananthakrishnan A.N. (2015). Epidemiology and risk factors for IBD. Nat. Rev. Gastroenterol. Hepatol..

[21] Molodecky N.A., Kaplan G.G. (2010). Environmental risk factors for inflammatory bowel disease. Gastroenterol. Hepatol..

[22] Spor A., Koren O., Ley R. (2011). Unravelling the effects of the environment and host genotype on the gut microbiome. Nat. Rev. Microbiol..

[23] Ng S.C., Bernstein C.N., Vatn M.H., Lakatos P.L., Loftus E.V., Tysk C., O’Morain C., Moum B., Colombel J.F. (2013). Geographical variability and environmental risk factors in inflammatory bowel disease. Gut.

[24] Kho Z.Y., Lal S.K. (2018). The human gut microbiome—A potential controller of wellness and disease. Front. Microbiol..

[25] Crevel R.W.R., Pickup J. (2006). Too clean, or not too clean: The Hygiene Hypothesis and home hygiene Clinical and Experimental Allergy. Clin. Exp. Allergy.

[26] Rodríguez J.M., Murphy K., Stanton C., Ross R.P., Kober O.I., Juge N., Avershina E., Rudi K., Narbad A., Jenmalm M.C. (2015). The composition of the gut microbiota throughout life, with an emphasis on early life. Microb. Ecol. Health Dis..

[27] Fouhy F., Ross R.P., Fitzgerald G., Stanton C., Cotter P.D. (2012). Composition of the early intestinal microbiota:Knowledge, knowledge gaps and the use of high-throughput sequencing to address these gaps. Gut Microbes.

[28] Milani C., Duranti S., Bottacini F., Casey E., Turroni F., Mahony J., Belzer C., Delgado Palacio S., Arboleya Montes S., Mancabelli L. (2017). The First Microbial Colonizers of the Human Gut: Composition, Activities, and Health Implications of the Infant Gut Microbiota. Microbiol. Mol. Biol. Rev..

[29] Roger L.C., McCartney A.L. (2010). Longitudinal investigation of the faecal microbiota of healthy full-term infants using fluorescence in situ hybridization and denaturing gradient gel electrophoresis. Microbiology.

[30] Schwartz S., Friedberg I., Ivanov I.V., Davidson L.A., Goldsby J.S., Dahl D.B., Herman D., Wang M., Donovan S.M., Chapkin R.S. (2012). A metagenomic study of diet-dependent interaction between gut microbiota and host in infants reveals differences in immune response. Genome Biol..

[31] Fallani M., Amarri S., Uusijarvi A., Adam R., Khanna S., Aguilera M., Gil A., Vieites J.M., Norin E., Young D. (2011). Determinants of the human infant intestinal microbiota after the introduction of first complementary foods in infant samples from five European centres. Microbiology.

[32] Fallani M., Young D., Scott J., Norin E., Amarri S., Adam R., Aguilera M., Khanna S., Gil A., Edwards C.A. (2010). Intestinal microbiota of 6-week-old infants across Europe: Geographic influence beyond delivery mode, breast-feeding, and antibiotics. J. Pediatr. Gastroenterol. Nutr..

[33] Arumugam M., Raes J., Pelletier E., Le Paslier D., Yamada T., Mende D.R., Fernandes G.R., Tap J., Bruls T., Batto J.M. (2011). Enterotypes of the human gut microbiome. Nature.

[34] Wu G.D., Chen J., Hoffmann C., Bittinger K., Chen Y.Y., Keilbaugh S.A., Bewtra M., Knights D., Walters W.A., Knight R. (2011). Linking long-term dietary patterns with gut microbial enterotypes. Science.

[35] Lozupone C.A., Stombaugh J.I., Gordon J.I., Jansson J.K., Knight R. (2012). Diversity, stability and resilience of the human gut microbiota. Nature.

[36] Mills S., Stanton C., Lane J.A., Smith G.J., Ross R.P. (2019). Precision nutrition and the microbiome, part I: Current state of the science. Nutrients.

[37] Hansen N.W., Sams A. (2018). The microbiotic highway to health—New perspective on food structure, gut microbiota, and host inflammation. Nutrients.

[38] Manzel A., Muller D.N., Hafler D.A., Erdman S.E., Linker R.A., Kleinewietfeld M. (2014). Role of “Western diet” in inflammatory autoimmune diseases. Curr. Allergy Asthma Rep..

[39] Esparza M.L., Sasaki S., Kesteloot H. (1995). Nutrition, latitude, and multiple sclerosis mortality: An ecologic study. Am. J. Epidemiol..

[40] Lauer K. (1994). The risk of multiple sclerosis in the U.S.A. in relation to sociogeographic features: A factor-analytic study. J. Clin. Epidemiol..

[41] Trompette A., Gollwitzer E.S., Yadava K., Sichelstiel A.K., Sprenger N., Ngom-Bru C., Blanchard C., Junt T., Nicod L.P., Harris N.L. (2014). Gut microbiota metabolism of dietary fiber influences allergic airway disease and hematopoiesis. Nat. Med..

[42] Koh A., De Vadder F., Kovatcheva-Datchary P., Bäckhed F. (2016). From dietary fiber to host physiology: Short-chain fatty acids as key bacterial metabolites. Cell.

[43] Den Besten G., Van Eunen K., Groen A.K., Venema K., Reijngoud D.J., Bakker B.M. (2013). The role of short-chain fatty acids in the interplay between diet, gut microbiota, and host energy metabolism. J. Lipid Res..

[44] Nastasi C., Candela M., Bonefeld C.M., Geisler C., Hansen M., Krejsgaard T., Biagi E., Andersen M.H., Brigidi P., Ødum N. (2015). The effect of short-chain fatty acids on human monocyte-derived dendritic cells. Sci. Rep..

[45] Hill C., Guarner F., Reid G., Gibson G.R., Merenstein D.J., Pot B., Morelli L., Canani R.B., Flint H.J., Salminen S. (2014). Expert consensus document: The international scientific association for probiotics and prebiotics consensus statement on the scope and appropriate use of the term probiotic. Nat. Rev. Gastroenterol. Hepatol..

[46] Kechagia M., Basoulis D., Konstantopoulou S., Dimitriadi D., Gyftopoulou K., Skarmoutsou N., Fakiri E.M. (2013). Health Benefits of Probiotics: A Review. ISRN Nutr..

[47] Ritchie M.L., Romanuk T.N. (2012). A meta-analysis of probiotic efficacy for gastrointestinal diseases. PLoS ONE.

[48] Sánchez B., Delgado S., Blanco-Míguez A., Lourenço A., Gueimonde M., Margolles A. (2017). Probiotics, gut microbiota, and their influence on host health and disease. Mol. Nutr. Food Res..

[49] Vieira S.M., Pagovich O.E., Kriegel M.A. (2014). Diet, microbiota and autoimmune diseases. Lupus.

[50] Thorburn A.N., Macia L., Mackay C.R. (2014). Diet, Metabolites, and “Western-Lifestyle” Inflammatory Diseases. Immunity.

[51] Ivanov I.I., Atarashi K., Manel N., Brodie E.L., Shima T., Karaoz U., Wei D., Goldfarb K.C., Santee C.A., Lynch S.V. (2009). Induction of Intestinal Th17 Cells by Segmented Filamentous Bacteria. Cell.

[52] Ochoa-Repáraz J., Mielcarz D.W., Wang Y., Begum-Haque S., Dasgupta S., Kasper D.L., Kasper L.H. (2010). A polysaccharide from the human commensal Bacteroides fragilis protects against CNS demyelinating disease. Mucosal Immunol..

[53] Baumgart D.C. (2017). Crohn’s Disease and Ulcerative Colitis: From Epidemiology and Immunobiology to a Rational Diagnostic and Therapeutic Approach.

[54] Rizzello F., Spisni E., Giovanardi E., Imbesi V., Salice M., Alvisi P., Valerii M.C., Gionchetti P. (2019). Implications of the westernized diet in the onset and progression of IBD. Nutrients.

[55] Scaioli E., Liverani E., Belluzzi A. (2017). The imbalance between N-6/N-3 polyunsaturated fatty acids and inflammatory bowel disease: A comprehensive review and future therapeutic perspectives. Int. J. Mol. Sci..

[56] Cabré E., Mañosa M., Gassull M.A. (2012). Omega-3 fatty acids and inflammatory bowel diseases-a systematic review. Br. J. Nutr..

[57] Wall R., Ross R.P., Fitzgerald G.F., Stanton C. (2010). Fatty acids from fish: The anti-inflammatory potential of long-chain omega-3 fatty acids. Nutr. Rev..

[58] Costantini L., Molinari R., Farinon B., Merendino N. (2017). Impact of omega-3 fatty acids on the gut microbiota. Int. J. Mol. Sci..

[59] Barbalho S.M., Goulart R.D.A., Quesada K., Bechara M.D., De Carvalho A.D.C.A. (2016). Inflammatory bowel disease: Can omega-3 fatty acids really help?. Ann. Gastroenterol..

[60] Vaarala O. (2012). Is the origin of type 1 diabetes in the gut?. Immunol. Cell Boil..

[61] Turck D. (2013). Cow’s milk and goat’s milk. World Rev. Nutr. Diet..

[62] Norris J.M. (2010). Infant and childhood diet and type 1 diabetes risk: Recent advances and prospects. Curr. Diabetes Rep..

[63] Åkerblom H.K., Vaarala O., Hyöty H., Ilonen J., Knip M. (2002). Environmental factors in the etiology of type 1 diabetes. Am. J. Med. Genet. Semin. Med. Genet..

[64] Esposito S., Bonavita S., Sparaco M., Gallo A., Tedeschi G. (2018). The role of diet in multiple sclerosis: A review. Nutr. Neurosci..

[65] Riccio P., Rossano R. (2015). Nutrition facts in multiple sclerosis. ASN Neuro.

[66] Jörg S., Grohme D.A., Erzler M., Binsfeld M., Haghikia A., Müller D.N., Linker R.A., Kleinewietfeld M. (2016). Environmental factors in autoimmune diseases and their role in multiple sclerosis. Cell. Mol. Life Sci..

[67] Monteiro R., Azevedo I. (2010). Chronic inflammation in obesity and the metabolic syndrome. Mediat. Inflamm..

[68] Saltiel A.R., Olefsky J.M. (2017). Inflammatory mechanisms linking obesity and metabolic disease. J. Clin. Investig..

[69] Ellulu M.S., Patimah I., Khaza’ai H., Rahmat A., Abed Y. (2017). Obesity & inflammation: The linking mechanism & the complications. Arch. Med. Sci..

[70] Makki K., Froguel P., Wolowczuk I. (2013). Adipose Tissue in Obesity-Related Inflammation and Insulin Resistance: Cells, Cytokines, and Chemokines. ISRN Inflamm..

[71] Saucillo D.C., Gerriets V.A., Sheng J., Rathmell J.C., MacIver N.J. (2014). Leptin Metabolically Licenses T Cells for Activation to Link Nutrition and Immunity. J. Immunol..

[72] Rai S.K., Fung T.T., Lu N., Keller S.F., Curhan G.C., Choi H.K. (2017). The Dietary Approaches to Stop Hypertension (DASH) diet, Western diet, and risk of gout in men: Prospective cohort study. BMJ.

[73] Rebholz C.M., Crews D.C., Grams M.E., Steffen L.M., Levey A.S., Miller E.R., Appel L.J., Coresh J. (2016). DASH (Dietary Approaches to Stop Hypertension) Diet and Risk of Subsequent Kidney Disease. Am. J. Kidney Dis..

[74] Hariharan D., Vellanki K., Kramer H. (2015). The Western Diet and Chronic Kidney Disease. Curr. Hypertens. Rep..

[75] Statovci D., Aguilera M., MacSharry J., Melgar S. (2017). The impact of western diet and nutrients on the microbiota and immune response at mucosal interfaces. Front. Immunol..

[76] Endo Y., Shoji T., Fukudo S. (2015). Epidemiology of irritable bowel syndrome. Ann. Gastroenterol..

[77] Lacy B.E., Patel N.K. (2017). Rome Criteria and a Diagnostic Approach to Irritable Bowel Syndrome. J. Clin. Med..

[78] Chey W.D., Kurlander J., Eswaran S. (2015). Irritable bowel syndrome: A clinical review. JAMA J. Am. Med. Assoc..

[79] Cozma-Petrut A., Loghin F., Miere D., Dumitrascu D.L. (2017). Diet in irritable bowel syndrome: What to recommend, not what to forbid to patients!. World J. Gastroenterol..

[80] Bardisi B.M., Halawani A.K.H., Halawani H.K.H., Alharbi A.H., Turkostany N.S., Alrehaili T.S., Radin A.A., Alkhuzea N.M. (2018). Efficiency of diet change in irritable bowel syndrome. J. Fam. Med. Prim. Care.

[81] Cancarevic I., Rehman M., Iskander B., Lalani S., Malik B.H. (2020). Is There a Correlation Between Irritable Bowel Syndrome and Lactose Intolerance?. Cureus.

[82] Dainese R., Casellas F., Mariné-Barjoan E., Vivinus-Nébot M., Schneider S.M., Hébuterne X., Piche T. (2014). Perception of lactose intolerance in irritable bowel syndrome patients. Eur. J. Gastroenterol. Hepatol..

[83] Cuomo R., Andreozzi P., Zito F.P., Passananti V., De Carlo G., Sarnelli G. (2014). Irritable bowel syndrome and food interaction. World J. Gastroenterol..

[84] Ford A.C., Lacy B.E., Talley N.J. (2017). Irritable bowel syndrome. N. Engl. J. Med..

[85] El-Salhy M., Hatlebakk J.G., Hausken T. (2019). Diet in Irritable Bowel Syndrome (IBS): Interaction with Gut Microbiota and Gut Hormones. Nutrients.

[86] Werlang M.E., Palmer W.C., Lacy B.E. (2019). Irritable Bowel Syndrome and Dietary Interventions. Gastroenterol. Hepatol..

[87] Tuck C.J., Muir J.G., Barrett J.S., Gibson P.R. (2014). Fermentable oligosaccharides, disaccharides, monosaccharides and polyols: Role in irritable bowel syndrome. Expert Rev. Gastroenterol. Hepatol..

[88] Marsh A., Eslick E.M., Eslick G.D. (2016). Does a diet low in FODMAPs reduce symptoms associated with functional gastrointestinal disorders? A comprehensive systematic review and meta-analysis. Eur. J. Nutr..

[89] Algera J., Colomier E., Simrén M. (2019). The dietary management of patients with irritable bowel syndrome: A narrative review of the existing and emerging evidence. Nutrients.

[90] Benninga M., Candy D.C.A., Catto-Smith A.G., Clayden G., Loening-Baucke V., Di Lorenzo C., Nurko S., Staiano A. (2005). The Paris Consensus on Childhood Constipation Terminology (PACCT) Group. J. Pediatr. Gastroenterol. Nutr..

[91] Forootan M., Bagheri N., Darvishi M. (2018). Chronic constipation: A review of literature. Medicine.

[92] Camilleri M., Ford A.C., Mawe G.M., Dinning P.G., Rao S.S., Chey W.D., Simrén M., Lembo A., Young-Fadok T.M., Chang L. (2017). Chronic constipation. Nat. Rev. Dis. Prim..

[93] Vriesman M.H., Koppen I.J.N., Camilleri M., Di Lorenzo C., Benninga M.A. (2020). Management of functional constipation in children and adults. Nat. Rev. Gastroenterol. Hepatol..

[94] Rao S.S.C., Sadeghi P., Batterson K., Beaty J. (2001). Altered periodic rectal motor activity: A mechanism for slow transit constipation. Neurogastroenterol. Motil..

[95] De Schryver A.M.P., Samsom M., Smout A.I.P.M. (2003). Effects of a meal and bisacodyl on colonic motility in healthy volunteers and patients with slow-transit constipation. Dig. Dis. Sci..

[96] Porter A.J., Wattchow D.A., Hunter A., Costa M. (1998). Abnormalities of nerve fibers in the circular muscle of patients with slow transit constipation. Int. J. Colorectal Dis..

[97] Suares N.C., Ford A.C. (2011). Systematic review: The effects of fibre in the management of chronic idiopathic constipation. Aliment. Pharmacol. Ther..

[98] Mahadeva S., Ford A.C. (2016). Clinical and epidemiological differences in functional dyspepsia between the East and the West. Neurogastroenterol. Motil..

[99] Drossman D.A. (2016). Functional gastrointestinal disorders: History, pathophysiology, clinical features, and Rome IV. Gastroenterology.

[100] Corsetti M., Fox M. (2017). The management of functional dyspepsia in clinical practice: What lessons can be learnt from recent literature?. F1000Research.

[101] Madisch A., Andresen V., Enck P., Labenz J., Frieling T., Schemann M. (2018). The diagnosis and treatment of functional dyspepsia. Dtsch. Arztebl. Int..

[102] Jung H.K., Talley N.J. (2018). Role of the duodenum in the pathogenesis of functional dyspepsia: A paradigm shift. J. Neurogastroenterol. Motil..

[103] Wilder-Smith C.H., Materna A., Wermelinger C., Schuler J. (2013). Fructose and lactose intolerance and malabsorption testing: The relationship with symptoms in functional gastrointestinal disorders. Aliment. Pharmacol. Ther..

[104] Tuck C.J., Biesiekierski J.R., Schmid-Grendelmeier P., Pohl D. (2019). Food intolerances. Nutrients.

[105] Junker Y., Zeissig S., Kim S.J., Barisani D., Wieser H., Leffler D.A., Zevallos V., Libermann T.A., Dillon S., Freitag T.L. (2012). Wheat amylase trypsin inhibitors drive intestinal inflammation via activation of toll-like receptor 4. J. Exp. Med..

[106] Filipović B.F., Randjelovic T., Kovacevic N., Milinić N., Markovic O., Gajić M., Filipović B.R. (2011). Laboratory parameters and nutritional status in patients with functional dyspepsia. Eur. J. Intern. Med..

[107] Elli L., Tomba C., Branchi F., Roncoroni L., Lombardo V., Bardella M.T., Ferretti F., Conte D., Valiante F., Fini L. (2016). Evidence for the presence of non-celiac gluten sensitivity in patients with functional gastrointestinal symptoms: Results from a multicenter randomized double-blind placebo-controlled gluten challenge. Nutrients.

[108] Carvalho R.V.B., Lorena S.L.S., De Souza Almeida J.R., Mesquita M.A. (2010). Food intolerance, diet composition, and eating patterns in functional dyspepsia patients. Dig. Dis. Sci..

[109] Santolaria S., Alcedo J., Cuartero B., Diez I., Abascal M., García-Prats M.D., Marigil M., Vera J., Ferrer M., Montoro M. (2013). Spectrum of gluten-sensitive enteropathy in patients with dysmotility-like dyspepsia. Gastroenterol. Hepatol..

[110] Akhondi-Meybodi M., Aghaei M.A., Hashemian Z. (2015). The role of diet in the management of non-ulcer dyspepsia. Middle East J. Dig. Dis..

[111] Pesce M., Cargiolli M., Cassarano S., Polese B., de Conno B., Aurino L., Mancino N., Sarnelli G. (2020). Diet and functional dyspepsia: Clinical correlates and therapeutic perspectives. World J. Gastroenterol..

[112] Vanheel H., Farré R. (2013). Changes in gastrointestinal tract function and structure in functional dyspepsia. Nat. Rev. Gastroenterol. Hepatol..

[113] Baumgart D.C., Carding S.R. (2007). Inflammatory bowel disease: Cause and immunobiology. Lancet.

[114] Hou J.K., Abraham B., El-Serag H. (2011). Dietary intake and risk of developing inflammatory bowel disease: A systematic review of the literature. Am. J. Gastroenterol..

[115] Reddavide R., Rotolo O., Caruso M.G., Stasi E., Notarnicola M., Miraglia C., Nouvenne A., Meschi T., De’ Angelis G.L., Di Mario F. (2018). The role of diet in the prevention and treatment of inflammatory bowel diseases. Acta Biomed..

[116] Rivière A., Selak M., Lantin D., Leroy F., De Vuyst L. (2016). Bifidobacteria and butyrate-producing colon bacteria: Importance and strategies for their stimulation in the human gut. Front. Microbiol..

[117] Wang Z.W., Ji F., Teng W.J., Yuan X.G., Ye X.M. (2011). Risk factors and gene polymorphisms of inflammatory bowel disease in population of Zhejiang, China. World J. Gastroenterol..

[118] Khalili H., Chan S.S.M., Lochhead P., Ananthakrishnan A.N., Hart A.R., Chan A.T. (2018). The role of diet in the aetiopathogenesis of inflammatory bowel disease. Nat. Rev. Gastroenterol. Hepatol..

[119] Castro F., De Souza H.S.P. (2019). Dietary composition and effects in inflammatory bowel disease. Nutrients.

[120] Dennis E.A., Norris P.C. (2015). Eicosanoid storm in infection and inflammation. Nat. Rev. Immunol..

[121] Fritsch J., Garces L., Quintero M.A., Pignac-Kobinger J., Santander A.M., Fernández I., Ban Y.J., Kwon D., Phillips M.C., Knight K. (2020). Low-Fat, High-Fiber Diet Reduces Markers of Inflammation and Dysbiosis and Improves Quality of Life in Patients With Ulcerative Colitis. Clin. Gastroenterol. Hepatol..

[122] Telle-Hansen V.H., Holven K.B., Ulven S.M. (2018). Impact of a Healthy Dietary Pattern on Gut Microbiota and Systemic Inflammation in Humans. Nutrients.

[123] Palafox-Carlos H., Ayala-Zavala J.F., González-Aguilar G.A. (2011). The Role of Dietary Fiber in the Bioaccessibility and Bioavailability of Fruit and Vegetable Antioxidants. J. Food Sci..

[124] Williams B.A., Grant L.J., Gidley M.J., Mikkelsen D. (2017). Gut fermentation of dietary fibres: Physico-chemistry of plant cell walls and implications for health. Int. J. Mol. Sci..

[125] Lobionda S., Sittipo P., Kwon H.Y., Lee Y.K. (2019). The role of gut microbiota in intestinal inflammation with respect to diet and extrinsic stressors. Microorganisms.

[126] Eswaran S., Muir J., Chey W.D. (2013). Fiber and functional gastrointestinal disorders. Am. J. Gastroenterol..

[127] Donnellan C.F., Yann L.H., Lal S. (2013). Nutritional management of Crohn’s disease. Ther. Adv. Gastroenterol..

[128] Hwang C., Ross V., Mahadevan U. (2012). Micronutrient deficiencies in inflammatory bowel disease: From A to zinc. Inflamm. Bowel Dis..

[129] Soares-Mota M., Silva T.A., Gomes L.M., Pinto M.A.S., Mendonça L.M.C., Farias M.L.F., Nunes T., Ramalho A., Zaltman C. (2015). High prevalence of vitamin A deficiency in Crohn’s disease patients according to serum retinol levels and the relative dose-response test. World J. Gastroenterol..

[130] Sun J. (2010). Vitamin D and mucosal immune function. Curr. Opin. Gastroenterol..

[131] Jørgensen S.P., Agnholt J., Glerup H., Lyhne S., Villadsen G.E., Hvas C.L., Bartels L.E., Kelsen J., Christensen L.A., Dahlerup J.F. (2010). Clinical trial: Vitamin D3 treatment in Crohn’s disease—A randomized double-blind placebo-controlled study. Aliment. Pharmacol. Ther..

[132] Dadaei T., Safapoor M.H., Aghdaei H.A., Balaii H., Pourhoseingholi M.A., Naderi N., Zojaji H., Azimzadeh P., Mohammadi P., Zali M.R. (2015). Effect of vitamin D3 supplementation on TNF-α serum level and disease activity index in Iranian IBD patients. Gastroenterol. Hepatol. Bed Bench.

[133] Kaitha S., Bashir M., Ali T. (2015). Iron deficiency anemia in inflammatory bowel disease. World J. Gastrointest. Pathophysiol..

[134] Siva S., Rubin D.T., Gulotta G., Wroblewski K., Pekow J. (2017). Zinc deficiency is associated with poor clinical outcomes in patients with inflammatory bowel disease. Inflamm. Bowel Dis..

[135] Sigall Boneh R., Sarbagili Shabat C., Yanai H., Chermesh I., Ben Avraham S., Boaz M., Levine A. (2017). Dietary Therapy With the Crohn’s Disease Exclusion Diet is a Successful Strategy for Induction of Remission in Children and Adults Failing Biological Therapy. J. Crohn’s Colitis.

[136] Shaikhkhalil A.K., Crandall W. (2018). Enteral Nutrition for Pediatric Crohn’s Disease: An Underutilized Therapy. Nutr. Clin. Pract. Off. Publ. Am. Soc. Parenter. Enter. Nutr..

[137] Comeche J.M., Caballero P., Gutierrez-Hervas A., García-Sanjuan S., Comino I., Altavilla C., Tuells J. (2019). Enteral nutrition in patients with inflammatory bowel disease. Systematic review, meta-analysis, and meta-regression. Nutrients.

[138] Nahidi L., Day A.S., Lemberg D.A., Leach S.T. (2012). Differential effects of nutritional and non-nutritional therapies on intestinal barrier function in an in vitro model. J. Gastroenterol..

[139] Miele E., Shamir R., Aloi M., Assa A., Braegger C., Bronsky J., De Ridder L., Escher J.C., Hojsak I., Kolaček S. (2018). Nutrition in Pediatric Inflammatory Bowel Disease: A Position Paper on Behalf of the Porto Inflammatory Bowel Disease Group of the European Society of Pediatric Gastroenterology, Hepatology and Nutrition. J. Pediatr. Gastroenterol. Nutr..

[140] Wall C.L., Gearry R.B., Day A.S. (2017). Treatment of Active Crohn’s Disease with Exclusive and Partial Enteral Nutrition: A Pilot Study in Adults. Inflamm. Intest. Dis..

[141] Gkikas K., Gerasimidis K., Milling S., Ijaz U.Z., Hansen R., Russell R.K. (2020). Dietary Strategies for Maintenance of Clinical Remission in Inflammatory Bowel Diseases: Are We There Yet?. Nutrients.

[142] Svolos V., Hansen R., Nichols B., Quince C., Ijaz U.Z., Papadopoulou R.T., Edwards C.A., Watson D., Alghamdi A., Brejnrod A. (2019). Treatment of Active Crohn’s Disease With an Ordinary Food-based Diet That Replicates Exclusive Enteral Nutrition. Gastroenterology.

[143] Halpin S.J., Ford A.C. (2012). Prevalence of symptoms meeting criteria for irritable bowel syndrome in inflammatory bowel disease: Systematic review and meta-analysis. Am. J. Gastroenterol..

[144] Andersen V., Olsen A., Carbonnel F., Tjønneland A., Vogel U. (2012). Diet and risk of inflammatory bowel disease. Dig. Liver Dis. Off. J. Ital. Soc. Gastroenterol. Ital. Assoc. Study Liver.

[145] Sigall-Boneh R., Pfeffer-Gik T., Segal I., Zangen T., Boaz M., Levine A. (2014). Partial enteral nutrition with a Crohn’s disease exclusion diet is effective for induction of remission in children and young adults with Crohn’s disease. Inflamm. Bowel Dis..

[146] Sigall-Boneh R., Van Limbergen J., Wine E. (2020). Dietary Therapies Induce Rapid Response and Remission in Pediatric Patients with Active Crohn’s Disease. Clin. Gastroenterol. Hepatol..

[147] Levine A., Sigall Boneh R., Wine E. (2018). Evolving role of diet in the pathogenesis and treatment of inflammatory bowel diseases. Gut.

[148] Levine A., Wine E., Assa A., Sigall Boneh R., Shaoul R., Kori M., Cohen S., Peleg S., Shamaly H., On A. (2019). Crohn’s Disease Exclusion Diet Plus Partial Enteral Nutrition Induces Sustained Remission in a Randomized Controlled Trial. Gastroenterology.

[149] Haskey N., Gibson D.L. (2017). An Examination of Diet for the Maintenance of Remission in Inflammatory Bowel Disease. Nutrients.

[150] Davis C., Bryan J., Hodgson J., Murphy K. (2015). Definition of the mediterranean diet: A literature review. Nutrients.

[151] Kaline K., Bornstein S.R., Bergmann A., Hauner H., Schwarz P.E.H. (2007). The importance and effect of dietary fiber in diabetes prevention with particular consideration of whole grain products. Horm. Metab. Res..

[152] D’Alessandro A., Lampignano L., De Pergola G. (2019). Mediterranean diet pyramid: A proposal for Italian people. A systematic review of prospective studies to derive serving sizes. Nutrients.

[153] Perez-Martinez P., Lopez-Miranda J., Blanco-Colio L., Bellido C., Jimenez Y., Moreno J.A., Delgado-Lista J., Egido J., Perez-Jimenez F. (2007). The chronic intake of a Mediterranean diet enriched in virgin olive oil, decreases nuclear transcription factor κB activation in peripheral blood mononuclear cells from healthy men. Atherosclerosis.

[154] Niewiadomski O., Studd C., Wilson J., Williams J., Hair C., Knight R., Prewett E., Dabkowski P., Alexander S., Allen B. (2016). Influence of food and lifestyle on the risk of developing inflammatory bowel disease. Intern. Med. J..

[155] Navarro P., Arias Á., Arias-González L., Laserna-Mendieta E.J., Ruiz-Ponce M., Lucendo A.J. (2019). Systematic review with meta-analysis: The growing incidence and prevalence of eosinophilic oesophagitis in children and adults in population-based studies. Aliment. Pharmacol. Ther..

[156] Kelly K.J., Lazenby A.J., Rowe P.C., Yardley J.H., Perman J.A., Sampson H.A. (1995). Eosinophilic esophagitis attributed to gastroesophageal reflux: Improvement with an amino acid-based formula. Gastroenterology.

[157] Arias A., González-Cervera J., Tenias J.M., Lucendo A.J. (2014). Efficacy of dietary interventions for inducing histologic remission in patients with eosinophilic esophagitis: A systematic review and meta-analysis. Gastroenterology.

[158] Kagalwalla A.F., Sentongo T.A., Ritz S., Hess T., Nelson S.P., Emerick K.M., Melin-Aldana H., Li B.U.K. (2006). Effect of six-food elimination diet on clinical and histologic outcomes in eosinophilic esophagitis. Clin. Gastroenterol. Hepatol. Off. Clin. Pract. J. Am. Gastroenterol. Assoc..

